# Differential proteostasis imbalance and the molecular basis of distinct synucleinopathies and tauopathies

**DOI:** 10.1098/rstb.2025.0272

**Published:** 2026-10-01

**Authors:** Virginie Redeker, Ronald Melki

**Affiliations:** ^1^ Laboratory of Neurodegenerative Diseases, CNRS, Paris, Île-de-France, France; ^2^ MIRcen, CEA, Fontenay-aux-Roses, Île-de-France, France

**Keywords:** proteostasis, Parkinson's disease, Alzheimer's disease, protein folding and aggregation, structural diversity, protein–protein interaction, alpha-synuclein, tau, neurodegenerative diseases, interactome

## Abstract

This short review discusses the structural and molecular events at the origin of diverse debilitating neurodegenerative diseases. The pathological consequences owing to the primary, secondary, tertiary and quaternary structural diversity of alpha-synuclein and tau proteins and the aggregates they form are presented. The crosstalk between alpha-synuclein and tau proteins aggregates structural heterogeneity and cellular homeostasis, and more precisely the proteostasis network is next considered. Overall, the proteostasis network appears as the master regulator of distinct synucleinopathies and tauopathies progression depending on its capacity to clear and/or disassemble to completion structurally diverse alpha-synuclein or tau fibrillar aggregates or not.

This article is part of the Theo Murphy meeting issue ‘ProteostaSys: a systems view of proteostasis’.

## 1. Structural diversity and dynamics of proteins

The structural diversity of the proteins our cells express defines the numerous key tasks and functions they execute. We indeed all know that from 100 000 to 1 000 000 proteins, depending on whether or not we consider alternative splicing, post-translational modification and internal ribosome entry sites, exerting different functions are encoded by our genome. The structure of over 78 860 human proteins has been solved from 1971 till the beginning of 2026 according to the Protein Data Bank (https://www.rcsb.org). A high degree of diversity, in relation to the function and/or reactions those proteins perform, is observed upon comparison of their structures. Indeed, 58 077 are asymmetric, 18 584 are cyclic, 2864 are dihedral, 36 are tetrahedral, 189 are octahedral, 147 are icosahedral and 413 are helical. Those proteins can further be classified based on their secondary structure content in all beta or alpha proteins (11 096 and 7066, respectively), alpha and beta single or multi-domain proteins (18 292), coiled coil proteins (231) and flexible/unstructured proteins (over 10 000).

The term flexible/unstructured proteins means that those proteins, in full or part, are highly dynamic, populating numerous transient conformations when they are pure [1,2]. In the presence of ligands ranging from low molecular weight molecules to lipid membranes, DNA or proteins, the folding landscapes those proteins explore are reduced. It is nonetheless important to bear in mind that the conformations a flexible/unstructured protein adopt depend on the ligand it binds [3]. Thus, flexible/unstructured protein ligands act as allosteric folding switches and their concentration and the affinity with which they are bound define the frequency of a given fold for an otherwise dynamic protein [4].

## 2. Why are flexible/unstructured proteins of such importance in synucleopathies and tauopathies?

The flexibility of a polypeptide chain is not the sole factor that drives aggregation as a significant number of highly structured proteins, ranging from antibody light and heavy chains to haemoglobin, can aggregate into pathogenic assemblies in a concentration-dependent manner. Nonetheless, the aggregation of a limited number of flexible/unstructured proteins is tightly associated with the most feared diseases: neurodegenerative diseases. The occurrence of these diseases is intimately linked to age, in other words, time. This suggests that populating conformations that are prone to interact with each other, yielding aggregates that are deleterious to the central and/or peripheral nervous system, is unfavourable, thus, dependent on time [5]. Alternatively, the probability of populating aggregation prone conformations may not be low but is counterbalanced by ligands that keep flexible/unstructured proteins from populating those conformations and subsequently from aggregation. Such ligands then act as master regulators for aggregation [6]. Indeed, the aggregation propensity of the protein tau, that is tightly involved in tauopathies such as Alzheimer's disease, depends on its inability to interact with its main partner, the protein tubulin, the building brick of microtubules. Similarly, the aggregation of alpha-synuclein, responsible for synucleinopathies like Parkinson's disease, depends on interactions with its main partner, cell membrane phospholipids. As the interactions of functional, monomeric tau and alpha-synuclein with their partners—whose diversity is not yet fully known—are dynamic, these proteins also interact with ligands ranging from nucleic acids and glycosaminoglycans to metal ions, that favour their aggregation [7–10]. The finding that a number of point mutations within the genes encoding proteins involved in neurodegenerative diseases leading to proteins where one amino acid is substituted by another, with similar or distinct properties, are associated with early onset familial forms of the diseases suggests that those mutations favour the formation of aggregation-prone conformations [2,11]. Similarly, when the mutation yields early termination of translation, the protein fragments thus generated may have, because of the absence of the rest of the protein, a much different aggregation propensity. It is nonetheless also possible that the conformations these point mutations favour, whether the substitutions are conservative or not, affect their interaction with ligands that keep these flexible/unstructured proteins from aggregating. Similarly, it is also understandable that the context of a polypeptide inclined to aggregate can modulate very significantly its aggregation propensity. Thus, it is impossible to distinguish between the two abovementioned processes controlling aggregation.

## 3. How do flexible/unstructured proteins aggregate?

Besides interacting with various ligands, flexible/unstructured proteins and/or polypeptide fragments of proteins, certainly in complexes with ligands, exhibit a high propensity to interact with themselves. The probability for a flexible/unstructured protein to interact with another to form a stable dimer, higher-order oligomers and aggregates is highly dependent on its concentration [12,13]. Indeed, it is only above a critical concentration—characteristic for each flexible/unstructured protein and/or protein fragments and dependent on the nature and number of interactions one protein establishes with another–that an aggregated form a flexible/unstructured protein and/or protein fragments is populated. When the multivalent intermolecular interactions between flexible/unstructured proteins in complex with their ligands are weak they yield dynamic condensates [14,15]. The latter process could be described by phase separation polymer-based physical models [16,17]. When the interactions are strong, driven by the formation of high numbers of hydrogen bonds between the main chains of the same polypeptide stretch in a beta strand conformation from different molecules, highly ordered fibrillar structures form [18,19]. These fibrillar assemblies can be considered as irreversible if their formation is not accompanied by an irreversible reaction that yields an inherent destabilization of the fibrils core [20]. Such fibrillar structures only exchange their constitutive units with those present in solution or in the cell through their ends. They grow if the cell senses a decrease in their physiological protein concentration upon aggregation and compensates by overexpressing them [21] and shrink [22,23] when their concentration decreases, for example if their expression is shut down upon aggregation.

## 4. Protein aggregation in neurodegenerative diseases

The aggregation of the flexible/unstructured proteins alpha-synuclein and tau plays a central role in a group of phenotypically diverse diseases named synucleinopathies and tauopathies [24,25], which together account for over 80% of neurodegenerative diseases. The global burden of these diseases has steadily increased since 1990 [26]. The aggregation of alpha-synuclein and tau occurs in different types of neurons, located in different regions of the peripheral and central nervous system [27,28]. Surprisingly, not all neurons containing aggregated alpha-synuclein and tau degenerate [29]. This means that while some neurons can cope with and maintain cellular homeostasis, in particular protein homeostasis (proteostasis), others cannot [12,30]. It is only the degeneration of specific neuron populations that results in different pathogenic phenotypes in the early stages of the different synucleinopathies and tauopathies [31,32]. This means that some neurons can survive despite the presence of aggregated alpha-synuclein and tau while others cannot. This remarkable observation reflects the diversity of neuronal cells. Indeed, neurons, not only across different brain regions but also within a given microscopic region, are highly microscopically and functionally heterogeneous [33]. This diversity is the consequence of differences in the proteomes they express, which underpin the distinct functions they perform ([34–38]; https://www.proteinatlas.org/humanproteome/brain/human+brain}regional_expression_within_the_brain). This may also be owing to the extent to which specific cellular processes, targeted by aggregated alpha-synuclein and tau, are solicited within certain neurons but not others [39,40]. For example, if the mitochondrial function is at its maximal capacity in one given population of neurons and is impaired by the aggregation alpha-synuclein or tau, while it is not in a neighbouring population of neurons, the latter population will be way more permissive to those aggregates as compared to a population with a very limited margin of adaptation.

## 5. The molecular roots of synucleinopathies and tauopathies diversity

The alpha-synuclein and tau proteins share no primary structure identity. Nevertheless, both proteins have the ability to adopt beta-strand secondary structure-rich conformations, often composed by beta strands held together through hydrogen bonds, electrostatic and hydrophobic intramolecular interactions between the side chains of amino acid residues connected by loop regions (figure 1A,C). Such stable tertiary structures are flat allowing highly ordered and complementary hydrogen bonds to form and the perfect stacking of these molecules into fibrillar assemblies (figure 1B,D). This stacking further stabilizes the beta-strand secondary structure-rich conformations. Within these assemblies, the hydrogen bonds between two consecutive strands are oriented parallel to the main fibril axis while the constituting beta strands are oriented perpendicular to the main fibril axis [19,41–43].

**Figure 1.**
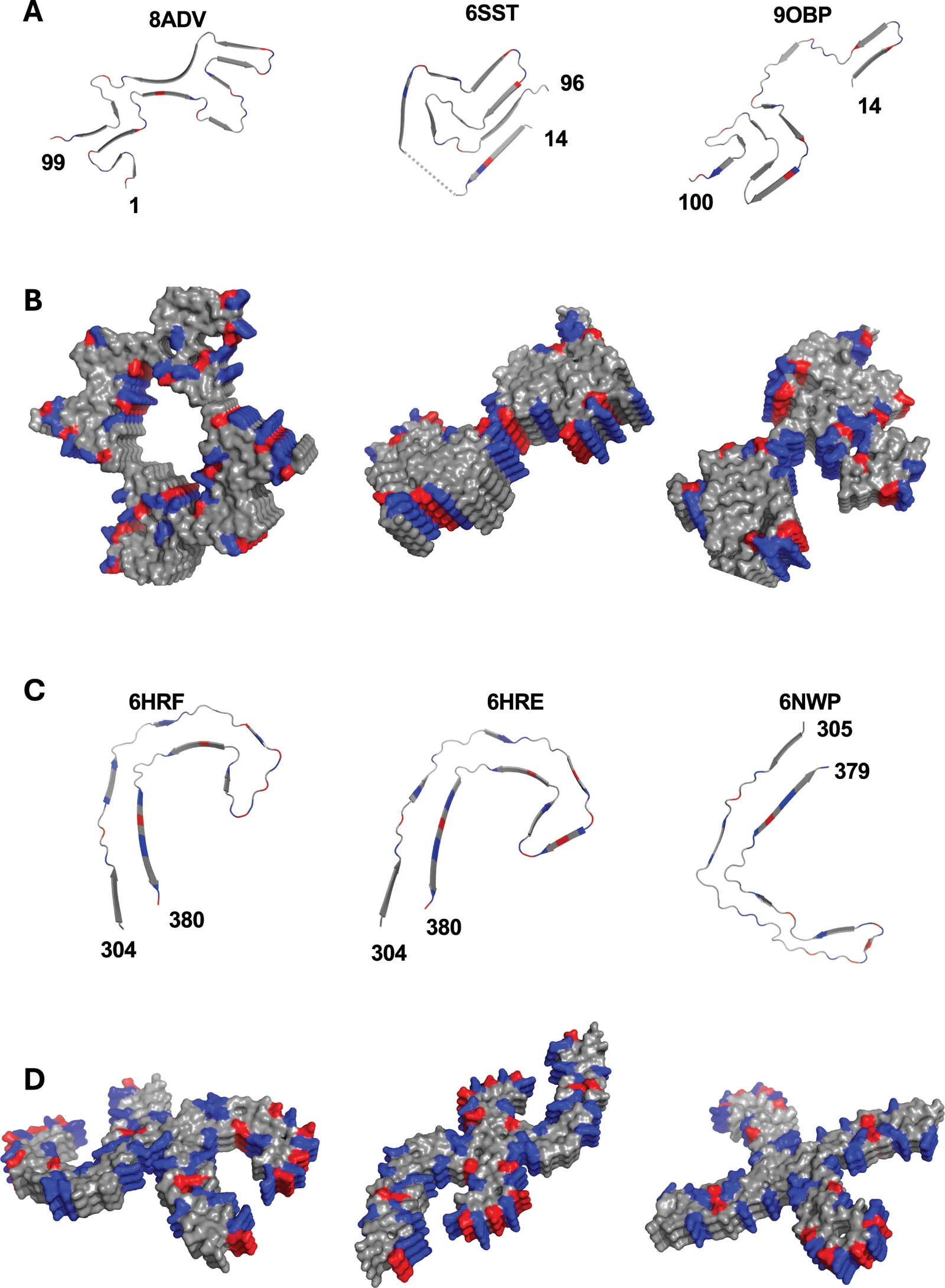
Structure of different amyloid forming folds alpha-synuclein and tau adopt in three different fibrillar polymorphs. (A) The main chain fold of the three different alpha-synuclein molecules with their Protein Databank (PDB) identity are shown. Basic residues are in blue, acidic residues in red and uncharged residues in grey. (B) Alpha-synuclein molecules, in the same fold and in a space fill representation, are piled up as they are in the fibrillar polymorphs they form. The view from top and side shows how flat each molecule layer is; how basic and acidic amino acid residues are exposed at the surface of the fibrils; how the stacking generates grooves, protrusions and arrays of acidic and basic amino acid residue side chains that run along the fibril surfaces that distinguish one polymorph from another; and finally how the two protofilaments constituting the three different polymorphs interact (apolar to polar interaction from left to right). (C) For tau protein, only the main chain fold within the core of three different fibrillar polymorphs (PDB identity indicated) is represented. The structure of the rest of the protein, surrounding the core (>80% of the amino acid residues), is unresolved. Basic residues are in blue, acidic residues in red and uncharged residues in grey. (D) Tau molecules, in the same fold and in a space fill representation, are piled up as they are in the fibrillar polymorphs they form. The view from top and side shows how basic and acidic amino acid residues are exposed within the core of the fibrils; how the stacking generates grooves, protrusions and arrays of acidic and basic amino acid residues side chains that distinguish one polymorph from another; and finally how the two protofilaments constituting the three different polymorphs interact through apolar interactions.

The intrinsic dynamics of alpha-synuclein and tau molecules allow different beta-strand-rich tertiary conformations in which distinct intramolecular interactions between amino acid side chains are established to form and disappear (see https://people.mbi.ucla.edu/sawaya/amyloidatlas/; https://amyloid-explorer.switchlab.org/database). Depending on their stability, these different conformers can stack to yield structurally distinct fibrillar assemblies (figure 1B,D). Point mutations associated with early onset forms of disease affect alpha-synuclein and tau molecule amino acid residue composition and therefore the tertiary structures or folds they populate (see https://people.mbi.ucla.edu/sawaya/amyloidatlas/; https://amyloid-explorer.switchlab.org/database). Their incomplete processing by the cellular clearance machinery also affects the tertiary structures they adopt (see https://people.mbi.ucla.edu/sawaya/amyloidatlas/; https://amyloid-explorer.switchlab.org/database). Furthermore, the intramolecular interactions within the folds alpha-synuclein and tau molecules populate are highly dependent on the chemical and physical environment (pH, viscosity, ionic strength, nature of ions, etc.) of those proteins. Thus, the conditions under which and/or the cellular compartments where alpha-synuclein and tau aggregate into fibrils favour different alpha-synuclein and tau tertiary structures or fold ensembles. Overall, the tertiary structures alpha-synuclein and tau molecules adopt define geometrically the way the molecules stack into fibrillar assemblies via highly complementary intermolecular hydrogen bonds (figure 1). It also determines the number of hydrogen bonds two consecutive molecules can establish. The tertiary structures alpha-synuclein and tau molecules adopt also define what amino acid side chains are exposed to the solvent at the surfaces of the stacks. This in turn determines their ability to interact laterally yielding fibrils made of multiple protofilaments as well as the size of the interface area through which two or more protofilaments interact (figure 1). The latter defines their nanomechanical properties, in particular their flexural rigidity [44–47]. Thus, fibril architecture and stability are both defined by the number of hydrogen bonds between two consecutive molecules within stacked alpha-synuclein and tau molecules and the lateral stacking of the protofilaments.

The diversity of the tertiary structures alpha-synuclein and tau populate, with sufficient stability to stack together and yield fibrillar assemblies, dictates not only their intrinsic architecture but also the surfaces they expose to their environment (figure 1A,C). The highly ordered array of hydrogen bonds, running parallel to the fibrils axis and constituting their scaffold, imposes limited freedom to amino acid residue stretches side chains protruding from the fibrils that do not exceed three amino acid residues. The latter are therefore highly ordered in space yielding longitudinal streaks of polar and non-polar amino acid side chains organized within topographical protrusions and grooves running parallel to the fibrillar axis (figure 1B,D). It is through these polar and non-polar streaks (coloured red and blue, for acidic or basic amino acid side chains, or grey for all other amino acid residues, respectively), resembling bar codes, that the fibrils interact with ligands ranging from small molecules to protein partners and cell components. The diversity of alpha-synuclein and tau molecules tertiary structures dictates the way they pile up through highly ordered arrays of hydrogen bonds running parallel to the fibrils. The latter yields a very large figure of amino acid side chains exposed at the stacks lateral surfaces, which in turn defines the protofilaments lateral stacking, the geometry of the protrusions and grooves running parallel to the fibrillar axis and their charges, in other words, the number of protofilaments constituting the fibrils, their topography and electrostatic properties, respectively (figure 1B,D). Thus, the diversity of these longitudinal streaks, running along the fibrils axis, defines how fibrillar alpha-synuclein and tau interact with their environment, in particular with cellular proteins.

## 6. Alpha-synuclein and tau aggregation in synucleinopathies and tauopathies, a gain or a loss of function?

The full range of alpha-synuclein and tau functions is mostly unknown as yet. It is widely accepted, nonetheless, that alpha-synuclein acts as an effector of lipid dynamics and a sensor of membrane curvature [48–55]. One specific cellular function attributed to alpha-synuclein upon interaction with membranes is N-ethylmaleimide sensitive factor attachment protein receptors (SNAREs) complex assembly and exocytosis, in particular, in synaptic vesicle turnover [56,57]. Similarly, the most documented functions for tau protein are those pertaining to its interaction with microtubules and the cell membranes, with consequences on the regulation of microtubule dynamics and stability, axonal transport, synaptic transmission and nuclear transport [58–60].

Knockout of alpha-synuclein and tau genes results in relatively subtle phenotypes. This certainly does not mean that these proteins are unimportant and is likely owing to redundancy associated with beta- and gamma-synuclein or different microtubule-associated proteins expression [61–65]. Nonetheless, the latter suggests that synucleinopathies and tauopathies are not simply the consequence of the loss of native alpha-synuclein and tau proteins functions upon aggregation.

Since synucleinopathies and tauopathies are not owing to the loss of the one or several functions alpha-synuclein and tau fulfil, they must be owing to a gain of pathogenic function [66,67]. The gain of toxic function of aggregated alpha-synuclein and tau was initially attributed to their ability to compromise/permeabilize the plasma membrane and/or intracellular membranous compartments. While those aggregates can occasionally breach lipid vesicles *in vitro*, they most likely alter membranous compartments integrity through interactions and redistribution of important membrane proteins [68–70]. Evidence for perturbation of important membrane protein dynamics and redistributions, leading possibly to the formation of novel signalling platforms, have indeed been reported. Alpha-synuclein and tau fibrils have been shown to interact and redistribute different ensembles of neuron membrane proteins that are partly overlapping. As described above, this differential interaction is owing to the fibrils topography and electrostatic properties. As different alpha-synuclein or tau fibrillar polymorphs, despite being made of the same protein, expose different surfaces to the solvent, it is very likely that they interact with partly overlapping ensembles of cellular proteins. This has major consequences as detailed below.

In addition to the acquisition of deleterious properties ranging from sequestration of important cellular proteins to their redistribution, the fibrillar forms of alpha-synuclein and tau gain the ability to exhibit differential resistance to the cellular clearance machinery. Those fibrils that resist best accumulate within cells until the burden is such that the cell dies and releases its content into the extracellular space. They then bind to unaffected cell membranes, compromising their integrity, and may be taken up with the consequences described above, in particular their growth/multiplication through the recruitment of the native form of their constituent proteins from the naive cell reservoir. Alternatively, or in parallel, they can be secreted from affected cells and taken up by unaffected cells or transferred from cell to cell within membranous structures. They acquire, thus, the ability to spread in a ‘prion-like’ manner and propagate the damage they cause across interconnected brain regions [71–73].

## 7. Alpha-synuclein and tau aggregates structure–pathology relationship

The probability with which the different alpha-synuclein or tau tertiary structures with aggregation propensity or compatible with seeds elongation are populated defines the rate at which aggregation occurs. Consequently, the different folds alpha-synuclein or tau populate determine the speed at which distinct synucleinopathies and tauopathies progress with time, in particular, when those aggregates resist the cellular clearance machinery. The different tertiary structures that alpha-synuclein or tau adopt are also highly dependent on their environment, as indicated above, including the tissue type, the cell nature and the cellular compartment where aggregation occurs. Furthermore, the lateral surfaces of alpha-synuclein or tau protofilaments within their fibrillar forms determine their capacity to bundle, thus masking proteolytic cleavage sites, which in turn defines their resistance to clearance by the cellular degradation machineries. In addition, because of the different surfaces they expose, structurally distinct fibrillar alpha-synuclein or tau polymorphs can redistribute and/or sequester into a non-functional state common but also different ensembles of partner proteins, thus affecting diverse cellular processes to different extents [74–77]. Structurally distinct alpha-synuclein and tau fibrillar polymorphs also exhibit differential tropism for neuronal cells as the presence of common or polymorph-specific receptors and their density at the surface of neuronal cells—e.g. different neurons, astrocytes, microglia, oligodendrocytes, pericytes, circulating immune cells and blood vessel endothelial cells—define whether these polymorphs can bind to a given cell type [68,78]. Finally, the amino acid side chains exposed at the surfaces of structurally distinct alpha-synuclein or tau fibrillar polymorphs also define whether they may be post-translationally modified so that they can be recognized by the cellular clearance machinery and as a consequence their post-translational modifications-dependent interactomes [79,80]. Altogether, this suggests a tight dependence of the pathological phenotype on both the structure and the interactome of fibrillar alpha-synuclein or tau.

A defined fibrillar alpha-synuclein polymorph interacts upon dilution in neurons or astrocytes lysates with different although in part overlapping ensembles of proteins as shown by pull down and identification of their partners using proteomic approaches and mass spectrometry identification (figure 2A; [68,78]). This likely results from the differential expression of those partner proteins (https://www.proteinatlas.org/ENSG00000104142-VPS18). For those proteins that are membranous, their presence, identity and density, in particular at the surface of neuronal cells, define whether fibrillar alpha-synuclein can bind to a given cell and the tropism of the fibrils towards those cells. Similarly, different fibrillar alpha-synuclein polymorphs interact with distinct ensembles of proteins upon dilution in neuron lysates [81]. This very likely defines the tropism of structurally distinct fibrillar alpha-synuclein towards different neuronal cells. The same figure applies to tau fibrils in neuron or astrocyte lysates [70]. This shows that structurally distinct fibrils made of alpha-synuclein or tau target different neuronal cell populations and compromise different cellular processes, thus, leading to diverse pathological phenotypes.

**Figure 2.**
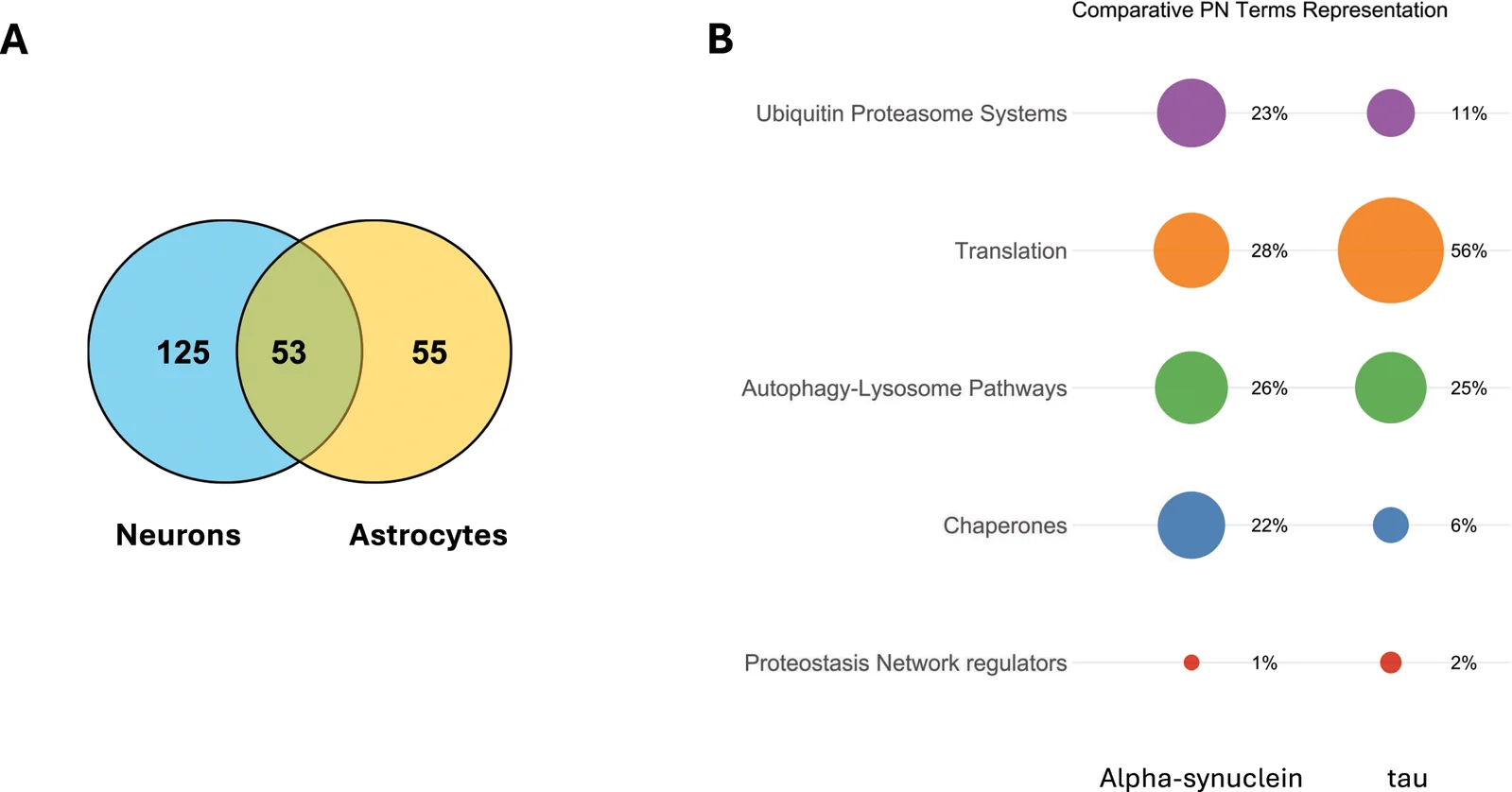
Differential interactomes of alpha-synuclein and tau at the cell membrane and within the proteostasis network. (A) Venn diagram of neuron and astrocyte membrane proteins that interact with one given alpha-synuclein fibrillar polymorph. The proteins were identified by pull-down experiments and mass spectrometry measurements. The full list is given in Shrivastava *et al.* [68]. A total of 178 neuron and 108 astrocyte membrane proteins were found to interact with exogenous alpha-synuclein fibrils upon their addition to neurons and astrocytes. Only 53 proteins are common. Thus, the interactome of fibrillar alpha-synuclein at the surface of distinct cells from the CNS is different. This may account for differential tropism. (B) A total of 33% of the proteins that bind to alpha-synuclein (left) and tau (right) fibrils belong to the proteostasis network (https://www.proteostasisconsortium.com/pn-annotation/). The relative weight of each proteostasis network component is represented for alpha-synuclein and tau fibrils. This shows that the ubiquitin proteasome system (UPS) and chaperones component of the proteostasis network are more involved in handling alpha-synuclein fibrils as compared to their tau counterparts. The opposite figure is observed for the translation component of the proteostasis network.

## 8. Proteostasis and the aggregation of alpha-synuclein and tau

The integrated network that encompasses ribosome-driven protein synthesis, protein folding mediated by highly selective or promiscuous molecular chaperones, protein addressing through signal peptides, molecular chaperones or carrier proteins to the appropriate cellular compartment and quality control by the ubiquitin proteasome system (UPS), autophagy and the lysosomal machinery, collectively maintains a balanced proteostasis in healthy cells ([82–84]; https://www.proteostasisconsortium.com/pn-annotation/). When protein synthesis, folding, trafficking or degradation are imbalanced, cells—in particular, those that are non-mitotic, such as neurons-—experience stress to different extents. Consequently, imbalanced or dysfunctional cellular proteostasis is tightly associated with accelerated ageing, tumourigenesis and apoptosis, metabolic and inflammatory diseases and neurodegeneration [85,86].

The identification of pathogenic alpha-synuclein and tau interactomes is widely considered as essential for understanding synucleinopathies and tauopathies. Indeed, structurally distinct alpha-synuclein or tau aggregates imbalance proteostasis by sequestering and/or redistributing important proteins within neurons membranous compartments or the cytosol, thereby mimicking their loss of function. The proxisome of pathogenic alpha-synuclein within the brains of patients affected by the three major synucleinopathies (Parkinson's disease (PD), Dementia with Lewy Bodies (DLB) and Multiple System Atrophy (MSA)) has been documented using proximity labelling assays [74,76]. Among the 261 proteins that preferentially interact with pathogenic phosphorylated alpha-synuclein, 16 belong to the autophagy-lysosome pathway, four to the UPS, five to translation regulation and six are molecular chaperones based on the proteostasis consortium annotation (https://www.proteostasisconsortium.com/pn-annotation/). Similarly, within the interactome of pathogenic phospho-tau in human Alzheimer's disease brain using immuno-affinity purification of phospho-tau associated proteins and mass spectrometry [87], 322 out of 1194 proteins, corresponding to 27% of the identified proteins, belong to the proteostasis network (141 belong to the autophagy-lysosome pathway, 95 to the UPS, 74 to translation regulation and 125 are molecular chaperones; https://www.proteostasisconsortium.com/pn-annotation/). While essential, these studies do not provide insight into the relationship between specific alpha-synuclein or tau fibrillar polymorphs and the differential sequestration and/or redistribution of membranous and cytosolic neuronal proteins. We therefore implemented a limited proteolysis coupled to mass spectrometry approach to identify putative interactors of structurally distinct alpha-synuclein fibrillar polymorphs derived from patients in human neuroblastoma cell lysates. A total of 19 E1, E2 and E3 ligases, eight deubiquitinating enzymes, 29 proteases and 37 molecular chaperones among which members from the Hsp40, CCT/TriC, 70 and 90 families, using highly stringent selection criteria (fold change (FC) > 2, *q*-value < 0.05), were identified among the 524, 238 and 453 putative interactors of alpha-synuclein fibrils derived from PD, DLB and MSA patient brains [81]. Similarly, we pulled down approximately 1500 and 350 protein binders after diluting alpha-synuclein or tau fibrils in Induced Pluripotent Stem Cell (iPSC)-derived human cortical neuron lysates, respectively. While the proportion of proteins belonging to the proteostasis network database within fibril binders was about 33%, the relative weight of each proteostasis network component varied significantly (figure 2B). Human neuroblastoma and iPSC-derived human cortical neurons compensate members of the proteostasis network sequestration by fibrillar alpha-synuclein. We indeed showed the differential protection and upregulation of 26 E1, E2 and E3 ligases, seven deubiquitinating enzymes, 39 molecular chaperones, two ubiquitin binding proteins and up to 14 proteasome subunits, using the selection criteria (FC > 2, *q*-value < 0.05), upon uptake of structurally distinct alpha-synuclein fibrillar polymorphs derived from PD, DLB and MSA patients [81]. Altogether, our recent analyses show that alpha-synuclein and tau aggregates exhibit structural characteristics that are differentially recognized by molecular chaperones and cellular quality control systems in charge of either disaggregating and/or refolding them or processing and clearing them within cells and immune cells in the extracellular space [81]. Thus, the molecular chaperones network becomes differentially solicited both by homeostatic protein synthesis and folding, the synthesis and folding of proteins bound to and sequestered by alpha-synuclein and tau aggregates, while at the same time being actively involved in those protein aggregates disassembly. The same figure applies to the UPS. Molecular chaperone networks and the UPS possess a finite capacity as demonstrated by proteostasis collapse upon cellular stress [88] or its challenge through the expression of aggregation-prone proteins in tissues expressing metastable but otherwise well-behaved proteins [89]. Thus, the additional challenge/workload these aggregates bring to the proteostasis network, the ground of healthy cellular maintenance, leads to its saturation/collapse and subsequent neuronal degeneration. As indicated previously, the different proteomes distinct neurons and other neuronal cell types express is at the origin of their high degree of functional diversity and specialization [83]. Those that exhibit sufficient resilience can cope with alpha-synuclein and tau aggregates and survive, while those that cannot, disappear.

## 9. Proteostasis, the master regulator of synucleinopathies and tauopathies

 Because of their distinct intrinsic structures and surfaces, conformationally different alpha-synuclein or tau fibrils interact differently with the components of the proteostasis machinery. While some polymorphs bind with high-affinity molecular chaperone machineries involved in their disaggregation—such as HSC70, its J domain co-chaperones and nucleotide exchange factors—others do not. The latter polymorphs are therefore not subject to chaperone-mediated disaggregation. Their clearance path is limited to autophagy and the lysosomal machinery [90–92]. When incompletely degraded by those paths, those fibrillar polymorphs either accumulate within neurons contributing further to neuronal stress, spread from affected to unaffected neurons [71] or activate the guardians of the central or peripheral nervous system integrity, namely astrocytes, microglia and circulating immune cells, leading to an immune response and inflammation [93–95]. By contrast, alpha-synuclein or tau fibrillar polymorphs that exhibit significant susceptibility to disaggregation may either be fully or partially disaggregated by the Hsc70 chaperone machinery. While full disaggregation and additional clearance by the UPS restore neuronal proteostasis, partial disaggregation may be highly deleterious. It yields fibrillar fragments that cannot be further degraded by the UPS with increased seeding propensity as the latter highly depends on fibril ends. Such short fibrils may exhibit a greater ability to spread from one neuron to another. They may further contribute to activation of the immune response and inflammation. Evidence for all of these figures exists [96–99].

Altogether, the proteostasis machinery through its ability, or failure, to handle structurally diverse alpha-synuclein and tau fibrillar aggregates acts as the master regulator of neurodegenerative processes. It either slows down synucleinopathies and tauopathies progression by restoring neuronal integrity or contributes to disease progression through inefficient clearance of alpha-synuclein or tau aggregates and/or its propensity to multiply pathogenic seeds.

## Data Availability

This article has no additional data.

## Theme

One contribution of 20 to a Theo Murphy meeting issue ‘ProteostaSys: a systems view of proteostasis’.
